# Non-Invasive Disease Specific Biomarker Detection Using Infrared Spectroscopy: A Review

**DOI:** 10.3390/molecules28052320

**Published:** 2023-03-02

**Authors:** Kiran Sankar Maiti

**Affiliations:** 1 Max–Planck–Institut für Quantenoptik, Hans-Kopfermann-Straße 1, 85748 Garching, Germany; kiran.maiti@mpq.mpg.de; Tel.: +49-289-14054; 2 Lehrstuhl für Experimental Physik, Ludwig-Maximilians-Universität München, Am Coulombwall 1, 85748 Garching, Germany; 3 Laser-Forschungslabor, Klinikum der Universität München, Fraunhoferstrasse 20, 82152 Planegg, Germany

**Keywords:** non-invasive diagnostics, infrared spectroscopy, biofluid, volatile organic compound (VOC), biomarker, prostate cancer, cerebral palsy, helicobacter pylori, island of stability (IOS), standard operating procedure (SOP)

## Abstract

Many life-threatening diseases remain obscure in their early disease stages. Symptoms appear only at the advanced stage when the survival rate is poor. A non-invasive diagnostic tool may be able to identify disease even at the asymptotic stage and save lives. Volatile metabolites-based diagnostics hold a lot of promise to fulfil this demand. Many experimental techniques are being developed to establish a reliable non-invasive diagnostic tool; however, none of them are yet able to fulfil clinicians’ demands. Infrared spectroscopy-based gaseous biofluid analysis demonstrated promising results to fulfil clinicians’ expectations. The recent development of the standard operating procedure (SOP), sample measurement, and data analysis techniques for infrared spectroscopy are summarized in this review article. It has also outlined the applicability of infrared spectroscopy to identify the specific biomarkers for diseases such as diabetes, acute gastritis caused by bacterial infection, cerebral palsy, and prostate cancer.

## 1. Introduction

### 1.1. Role of Metabolites in Diagnosis

Many life-threatening diseases develop silently in the human body, and symptoms only appear when the diseases are already rich in their advanced stage. In this regard, cancer [1], heart diseases [2], stroke [3], chronic obstructive pulmonary disease (COPD) [4], diabetes [5], Alzheimer’s [6], etc., are the most vulnerable diseases and cause millions of deaths worldwide in every year. The primary cause of the high death rate of these diseases is the lack of diagnostic techniques in their early disease stage. In addition, most of the diagnostics are expensive and demand invasive sample collection (especially for cancer) which may create a physical risk as well as psychological stress [7,8]. For example, until now, tissue biopsy has been the only reliable diagnostic method for cancer. However, it is an invasive process; therefore, as a rule of thumb tissue biopsy is used only with definite symptomatic cases. Unfortunately, by the time of detection, the diseases are already in the advanced stage, and it is too late in many cases. Several attempts have been taken to develop non-invasive detection of cancer. For example, great efforts are being made to develop imaging methods for tumour detection [9,10]. Unfortunately, because of technical reasons, small tumours cannot be detected at their initial stage by imaging methods. This implies a possibility of realizing only advanced-stage diagnosis, giving a chance for cancer cells to be metastasized. Alternatively, metabolic analysis of biofluids already demonstrated its power to detect many diseases [11]. For example, many life-threatening diseases such as diabetes, Alzheimer’s, cardiovascular disease, stroke, schizophrenia, etc., are characterized by changes in metabolites in the biofluids [12]. The advantage of the metabolites-based diagnosis is that most of the biofluids can be collected by minimally invasive as well as non-invasive processes. Therefore, metabolites-based diagnosis has promising potential to be the future clinical method if the connection between disease and observed metabolites in biofluids can be established reliably. This is the reason why the main focus of many researchers is directed to investigate changes in the metabolic composition of human biofluids at the onset of the disease: blood, urine, breath, etc.

### 1.2. Source of Metabolites

Biochemical reactions are essential processes to keep cells alive [13,14]. Thousands of parallel and sequential biochemical reactions are taking place ceaselessly in the human body. Many biological molecules are produced as products or byproducts of these biochemical reactions [15]. In physiology, these biochemical products are called metabolites [16]. Many of these metabolites are redundant or even toxic to the cell. Therefore, it is necessary to remove them from the cell. Biofluids, which are constantly supplying reagents to each cell of the body to maintain the supply chain for biochemical reactions also act as carriers to remove the excess chemical products from the cell. Finally, these nonessential metabolites remove from the body by different forms of waste, e.g., urine, faeces, sweat, breath, etc. Since these biofluids and semi-solid bio-wastes contain metabolites, essentially, they carry information about the internal chemistry of the body. Therefore, by monitoring the metabolites composition of the bio-wastes, it is possible to understand the internal chemical processes of the body; as a matter of fact, the body state will be understood. In general, in the normal physical condition of any living being, all biochemical reactions are taking place in a controlled manner and the production of metabolites maintained an equilibrium condition [17]. In case of changes in lifestyle or disease, chemical processes in cells may change completely or their reaction rate may change. Therefore, the concentration of one or many metabolites deviates from their equilibrium state. By analysing the composition and/or concentrations of metabolites, one can try to recognize the disease at its early (asymptomatic) stage [17].

### 1.3. The Current State of Metabolic Analysis

Metabolites are highly dynamic in size. The dynamic range of the size of metabolites varies from diatomic molecule to poly-atomic molecule consisting of thousands of atoms. Several hundreds of metabolites are already identified in human biofluids and many of their metabolic pathways are uncovered [18]. A great effort has been devoted to establishing the connection between metabolites and diseases [19]. Significant success has been achieved for large-size metabolites. Many of them are routinely used for clinical diagnosis. For example, blood sugar is used to determine the diabetic state of a person [20,21], prostate-specific antigen (PSA) is used for prostate cancer screening [22], etc. However, these diagnostics are mainly based on blood serum analysis, which is a minimally invasive process. Moreover, only after a significant change in the concentration, do those metabolites indicate the presence of the disease, in many cases, by that time, diseases are already in the advanced stage.

### 1.4. Volatile Organic Compounds (VOCs)

Recently smaller-size metabolites appear as the most promising approaches in the field of metabolomics for the diagnosis of diseases [23,24,25]. Typically, a molecular weight under 200 *Dalton* is considered a small metabolite. Usually, these molecules have high vapour pressure at low temperatures and easily reach the gaseous state. Commonly, these molecules are called volatile organic compounds (VOCs). One of the main sources of human VOCs is the exhaled breath which can be collected completely non-invasively. Other common sources of human VOCs are the headspace of urine, blood, saliva, faeces, etc. In terms of collection and handling, the samples of breath and urine may be the most appropriate because both biofluids are necessary to release from the human body at regular intervals of time. Therefore, there is no physical and/or mental stress in the sample collection processes. These make these two biofluids attractive bio-probes for the development of non-invasive diagnostics. In this review, the development of infrared spectroscopy based a fully non-invasive diagnostics is discussed.

## 2. Detection Techniques for VOCs

### 2.1. Mass-Spectroscopy

Diagnosis of disease by smelling the body is a practice from the ancient age; however, it was never been studied scientifically until recent years [26]. Recently it was reported that sniffer dogs were able to detect malignant tumours [27,28]. First, it was noticed by a 44-years old woman when her dog was constantly sniffing on a mole on her left thigh; however, the dog did not have any interest in other moles. This particular mole was excised and histological examination confirmed malignant melanoma. Williams et al. concluded in their report in The Lancet “Perhaps malignant tumours such as melanoma, with their aberrant protein synthesis, emit unique odours which though undetectable to man, are easily detected by dogs with their well-developed rhinencephalon”. This observation and report are very promising; however, in the twenty-first-century diagnosis by dogs can not be a reliable technique for cancer detection. To my knowledge, Linus Pauling, the American physicist, first reported a scientifically systematic study of VOCs by gas–liquid partition chromatography [29]. Along with his colleagues, he identified and reported around two hundred VOCs. Up to now, more than three thousand VOCs have been identified [26,30]. Practically, most of them were identified by different mass-spectrometry methods, e.g., gas chromatography–mass spectrometry (GC-MS) [31], ion mobility spectrometry (IMS) [32,33], proton transfer reaction mass spectrometry (PTR-MS) [34], selected ion flow tube mass spectrometry (SIFT-MS) [35], etc. Mass spectrometry has a fairly high sensitivity at the level of 100 ppt (parts-per-trillion) [36]; however, due to the complex and not completely controlled process of the sample preparation, it suffers from poor accuracy and as a result does not show reproducibility in VOC analysis. This makes a statistical set of data unreliable [37]. An indirect confirmation of this statement is the insufficient accuracy of the corresponding diagnostics (the benchmark is accuracy above 90%), revealed after analysing the literature data for a very wide range of diseases. The accuracy becomes higher by using one GC-MS instrument and one operator throughout the study [38]. This statement makes a question mark over the reliable use for the medical diagnosis. Moreover, mass spectrometers are very expensive and bulky in size.

### 2.2. e-Nose and QEPAS

Another developing instrument, the so-called electronic nose (e-nose) [39,40] is an attractive experimental tool, especially concerning the cost and size of the instrument. It uses a series of chemical sensor arrays to mimic the human smelling system. Pattern recognition using machine learning makes the e-nose a user-friendly technique for the recognition of VOCs in real-time. A nano structure-based e-nose device is extremely small in size [41]. Additionally, the e-nose sensors are more cost-effective than any other existing VOC detection technique. However, being a “black box”, different results were obtained by different research groups with e-nose; moreover, could not reveal metabolites [42]. Therefore, more investigation is necessary before its introduction into clinical application as a supplementary diagnostic technique. Recently developed quartz-enhanced photoacoustic spectroscopy (QEPAS) [43,44] for multi-gas detection holds a lot of promise to be a diagnostic tool; however, a proper investigation is required to carefully check the applicability in clinical diagnosis.

### 2.3. Infrared Spectroscopy

Compared to MS and e-nose, infrared spectroscopy represents the most fundamental technique for the detection and identification of molecules. Infrared spectroscopy is well established as a powerful and widely used tool for the analysis of molecular structure and dynamics in the fields of chemistry and physics [45,46,47,48,49]. It uses molecular vibrations as a probe to identify the molecule by structural analysis [50,51,52,53,54,55,56,57]. In practice, infrared light is used to excite molecular bonds and measure the absorption of light during their vibrations. Each chemical bond has a unique vibrational energy [58,59]; therefore, they absorb a specific wavelength of infrared light. As a result, each molecule produces a set of unique spectral features in the acquired infrared spectra. The set of unique spectral features from a particular molecule is called a fingerprint of the molecule [60]. The position, strength, and shape of the molecular fingerprint are used to identify and quantify a molecule from a mixture of molecules in biofluids [61,62,63].

### 2.4. Application of Infrared Spectroscopy to Biofluids and Tissue

As mentioned in the previous subsection, infrared light excites the molecular bonds only; therefore, molecules remain unperturbed. Additionally, the unique behaviour of each molecular bond allows for label-free extraction of biochemical information from biofluids and tissues. Particularly these two properties of infrared spectroscopy make it an attractive tool for biomarker-based early disease diagnostics. For example, a blood-based cancer diagnosis by infrared spectroscopy is a rapidly expanding research area [64]. A multi-institutional study of infrared fingerprints of plasma and serum samples for different cancer types demonstrated a promising result to identify different cancer types [65]. The infrared spectroscopic method was also employed for the diagnosis of diseases such as type 2 diabetes [66], HIV [67], etc., by blood sample analysis. In a recent study, salivary vibrational modes were analysed by attenuated total reflection–Fourier transform infrared (ATR-FTIR) spectroscopy to distinguish between healthy and COVID-19 patients [68]. As a biofluid, urine is also analysed by FTIR spectroscopy for the diagnosis of different renal diseases [63] and diabetes [69]. FTIR imaging of tissue is the fastest diagnosis method for histopathology of breast cancer [70,71]. The success of infrared spectroscopy for the analysis of liquid biofluids and tissue naturally motivated researchers to apply the technique for gas-phase biofluid analysis. However, this powerful tool can not be used in a straightforward way, especially for the identification of VOCs from gaseous biofluids. A brief working principle, advantages, and limitations of infrared spectroscopy for VOC analysis are discussed in the following sections.

## 3. Infrared Spectroscopy of Gaseous Biofluids

The major obstacle of infrared spectroscopy for the analysis of biological samples is the large amount of water vapour contained in gaseous biofluids. For example, a breath sample of a healthy person contained 5–7% of water vapour in a normal condition [72,73]. Such high water contained not only absorbs a large amount of infrared intensity but also the absorption spectra of water practically cover the most important spectral region where many VOCs yield their fingerprints. As a result, spectral features of VOCs of lower concentration (trace amount) are buried under the water spectra. In these circumstances, infrared spectroscopy is practically paralysed to reveal most of the VOCs. Therefore, the primary task is to remove water vapour from the biofluids to make use of the utility of infrared spectroscopy. The recent progress on the water vapour suppression technique opens a new window to investigate volatile metabolites in gaseous biofluids [74]. A brief description of the water vapour suppression technique is presented in the following section.

**Figure 1:**
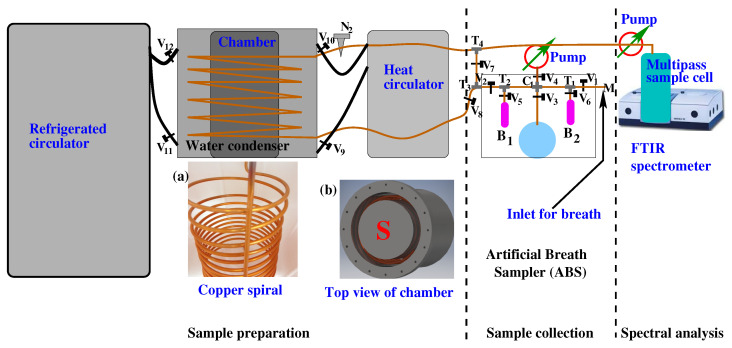
A schematic diagram of the experimental scheme for gaseous biofluid analysis by infrared spectroscopy. It consists of three major parts: (1) sample collection—in this part, breath or headspace of liquid biofluids is collected; (2) sample preparation—a water-suppressed sample is prepared for infrared spectroscopy when gaseous biofluids are passing through the “Water Condenser”; (3) spectral analysis—water suppressed gaseous sample is collected in a multipass gas cell and measured with an FTIR spectrometer. For details see the Ref. [74].

A schematic of the experimental scheme is presented in Figure 1. A detailed description of the experimental setup and working principle were presented in a previous publication [74]. There are three major units in the experimental setup, namely, (1) a sample collector, (2) a sample preparation unit, and (3) an infrared FTIR spectrometer. The sample collector system is designed in such a way that it is able to accept gaseous samples as well as the headspace of liquid biofluids [75]. Gaseous biofluid, e.g., breath is collected in a Tedlar bag or canister, and liquid biofluids, e.g., urine, blood, etc. are collected in a specially designed well-sealed glass flask. Before the sample injects into the sample collector, the complete system is evacuated by two vacuum pumps to remove any trace of contamination from the previous measurements. Breath samples or headspace of liquid biofluids are transferred to the empty sample collector by releasing the valve. Point to be noted that in the case of liquid biofluids, the flask needs to be partially filled by the biofluid and keeps it for a sufficient time in the flask to allow volatile compounds to be escaped from the liquid via sublimation. The VOCs are then accumulated on the top of the liquid as headspace. Unlike breath, the headspace sample collection aimed at further spectroscopic analysis still has no established standard operating procedure (SOP).

The biofluids collected from the test person often need to be stored temporarily. There are established protocols to store liquid biofluids; however, gaseous biofluid storage is under development [76,77]. One of the common storage devices for VOCs is the Tenax sorbent tube. The stability of VOCs trapped from breath samples in a Tenax adsorbent tube and stored −80 °C was studied over a year [78]. A significant loss was observed, and after 6 months only 27% of the sample was recovered. The study recommended the storage of VOCs for only 1.5 months. For infrared spectroscopy, sample storage by Tenax tube has yet not been tested. Breath samples were stored in Tedlar bags at 4 °C and tested carefully for several weeks. Approximately 30% drop of carbon monoxide was observed; however, bigger molecules such as methane, acetone, isoprene, etc., remain constant [74,79]. This observation was performed only for a few molecules. For the establishment of SOP, further investigation with a large number of VOCs is necessary. On the other hand, storage of breath samples using a Tedlar bag required significant storage space. Therefore, to optimize the storage capacity and reliability of the biomarker investigation, it is recommended to perform the measurement within a week.

A water condenser is a part of the experimental setup where water vapour is suppressed by condensation. It is a closed metal chamber containing a 12 m long, spiral copper tube, through which a gas-phase biofluid is transferred from the sample collector to a measurement cell attached to an FTIR spectrometer. The metal chamber in the water condenser is filled with a special liquid operating in a high dynamic temperature range between −95 °C and +45 °C. Before the gas-phase biofluid is allowed to transfer through the water condenser, the liquid is cooled down to −60 °C by a refrigerated circulator. At −60 °C, the sample is allowed to transfer through the spiral copper tube with a precisely controlled flow rate of 3 mL/s. A significant amount of water vapour is removed from the sample when it transfers through the cold copper tube. A reduction factor of above 2500 is achieved at −60 °C when the breath sample is transferred through a water condenser. Finally, the water-suppressed gas-phase biofluid is transferred to the multipass sample cell. After each experiment, the copper tube is cleaned by heating up the special liquid to 45 °C with a heat circulator and vacuum pumps. A detailed description of the setup and its working principle was reported in a separate article [74].

After the water suppression, the gaseous biofluid is transferred to a multipass cell which is placed inside the FTIR spectrometer. The infrared light travels approximately 4 m inside the cell. Infrared absorption spectra of biofluid are collected by a liquid nitrogen-cooled MCT detector in a range from 500 cm${}^{-1}$ to 4000 cm${}^{-1}$. Usually, two different types of experiments need to perform for the biofluids analysis. One included a single case aimed to find out metabolites present in the sample [75], and another aimed to find out the statistical difference between healthy volunteers and disease groups. For the first case, spectral data are analysed by the component analysis, and for the second case a statistical analysis is performed. In component analysis, the known molecular fingerprints are used for fitting the observed spectral features [80]. Statistical analysis is performed using unsupervised as well as supervised statistical methods.

## 4. Islands of Stability (IOS)

Identification and quantification of the metabolites for an individual have significant importance for the understanding of the internal chemistry of the body; however, it may have even greater importance for disease diagnosis if a reliable correlation among metabolites and disease can be found. Along with the reliable identification of metabolites, it is essential to monitor the dynamics of metabolite concentration over time, for the diagnosis of diseases. The existence of an individual metabolic phenotype (IMP) approach has been under discussion for several years [81,82,83]. Long-term stability has been reported for several biofluids [84,85]; however, until recent years, stability of the volatile metabolites in breath samples have been reported only for few days [77,86]. The first long-term study with several months has been reported recently. A few volatile metabolites in the human breath have been detected and quantified by infrared spectroscopy in a time period over eighteen months [17]. The observations are the following.

### 4.1. Effect of Physical Exercise

**Figure 2:**
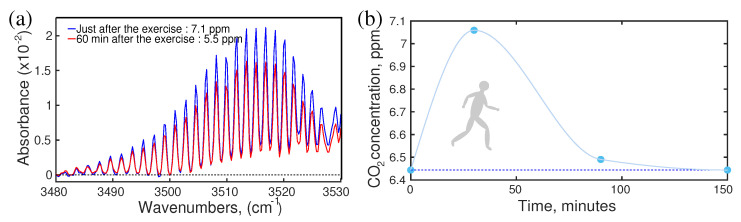
The effect of physical exercise. (**a**) Absorption spectra of breath CO${}_{2}$. (**b**) The dynamics of CO${}_{2}$ concentration during and after jogging. (Ref. [17]).

It is well-known that the concentration of the metabolite changes due to diet, lifestyle, sports, health conditions, etc. However, the question is what is the range of change in the concentration of the specific metabolites? There are many studies reported for each specific case. For example, during physical exercise breath components were measured and reported [87,88,89,90,91]; however, there was no information about the dynamics of metabolites concentration after exercise. Knowledge about the “after–effect” of any circumstance is crucial for a reliable diagnosis. A follow-up study of breath samples was reported recently. In this study, a healthy volunteer performed jogging for 30 min and donated breath samples “just before” and “just after” jogging and also follow-up samples for two hours. The infrared spectroscopic method has been used to monitor the concentration of VOCs. Many VOCs showed a change of concentration during jogging and gradually returned to their normal state in a short time period. As an illustration, the dynamics of carbon dioxide have been presented in Figure 2a,b. A sharp rise of CO${}_{2}$ concentration was observed with the increase in jogging time. After jogging, CO${}_{2}$ concentration dropped,;however, at a slower rate than CO${}_{2}$ concentration increased. Two hours after jogging, CO${}_{2}$ concentration returned to its normal state (Figure 2b). It was expected that similar behaviour would be observed for other kinds of physical activities. This information is important to develop the standard operating procedure (SOP) for breath sample collection.

### 4.2. Effect of Coffee Drinking

**Figure 3:**
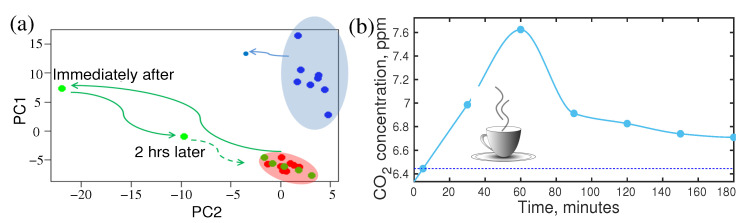
Effect of coffee for rare and moderate coffee drinkers. (**a**) The shift of the steady state point in PCA representation and its return for the rare coffee drinker (green) and a moderate drinker (blue). (**b**) The dynamics of CO${}_{2}$ concentration of a rare coffee drinker in time. Note that the effect reaches its maximum 1 h after the intake and lasts up to 7 h (evaluation). (Ref. [17]).

In metabolism, foods, and drinks may have the strongest and instant influences [88,92,93,94,95,96]. Therefore, it is expected that a strong effect of foods and drinks would be observed in the breath VOCs. Of course, the change in metabolic concentration is mild in the case of regular food and drinks; however, a strong effect is observed in the case of a new type of food or drinks. There is a classic example of the coffee effect reported recently [17]. Two healthy volunteers of similar age, one is a moderate coffee drinker (>5 cups/day) and another volunteer drinks coffee occasionally (1 cup/two weeks), took part in the study. Both of them used the same kind of coffee and provided breath samples at regular intervals of time, starting from “just before” drinking the coffee and until three hours after the coffee intake. The experiment was performed and the results were compared without any attention to the individual’s physical state or previous meal intake. Infrared spectral data were analysed by component analysis as well as statistical analysis, e.g., principal component analysis (PCA). Results are presented in Figure 3a,b. A maximum concentration variation of metabolites was observed about an hour later than coffee intake. The concentration of metabolites gradually decreased and returned to its steady state. The maximum shift of the steady state point and its return to the steady state reflects the characteristics of the two volunteers. In the case of the rare coffee drinker, the shift is significantly high and it took much longer time to return to a steady state in comparison to the moderate coffee drinker. This observation is expected since coffee is an unknown chemical for the rare coffee drinker’s body and it reacts strongly. This information is crucial to establish an SOP for the metabolic analysis of biofluids.

### 4.3. Effect of Fasting

**Figure 4:**
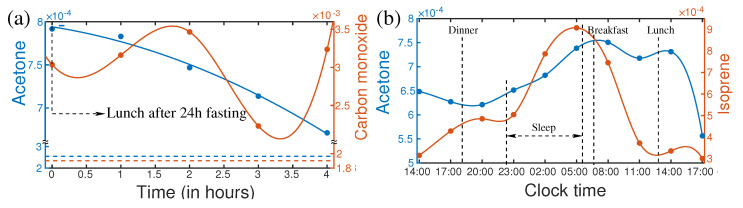
(**a**) Variations of isoprene and carbon monoxide after the first meal ending 24 h fasting (left). Horizontal lines are the steady-state levels of the corresponding VOCs. (**b**) Circadian variations of acetone and isoprene of a healthy person. (Ref. [17]).

Fasting is not a regular human activity; however, it is a part of our life. Especially in the modern lifestyle, people often skip one or two meals in a day. Fasting has a strong influence on the metabolic profile of the person [95,96,97]. As a consequence of fasting, the concentration of acetone changes significantly. In a recent publication, a follow-up investigation of breath metabolites after fasting was reported [17]. The “after–effect” of 27 h of fasting was monitored for four hours. Many metabolites showed a significant shift in their concentration level due to fasting. For example, the variation of concentration of acetone and carbon monoxide were plotted in Figure 4a. After 24 h of fasting, the breath acetone level of a healthy person increased by approximately three times. At the end of fasting, the volunteer had a normal meal. The acetone level started to drop and still remain two times higher than the normal level after 4 h of the meal, whereas only 20% variation of acetone was observed in a circadian cycle. Therefore, it is extremely important to know the last food intake before collecting the biofluids, especially for diagnostic purposes. Otherwise, an abnormal change in acetone concentration may mislead the diagnosis.

### 4.4. Circadian Variation of Metabolites in Breath

In the circadian cycle, human activities change in a large spectrum from sleeping to a high level of mental and physical activities. These may have strong influences on the concentration of metabolites in biofluids [98]. In general dynamics of metabolites are expected to follow the circadian rhythm. Knowledge of change of metabolite composition in the circadian cycle [98] is essential for the development of SOP for metabolic analysis. The variation of acetone and isoprene over 27 h for a healthy person are plotted in Figure 4b. A strong increase in isoprene concentration was observed during sleeping. After awakening, isoprene concentration dropped very fast until lunchtime and remained low till evening. Many supportive pieces of evidence suggest that isoprene is related to cholesterol biosynthesis [99,100]. Therefore, measurement of isoprene could potentially be used for lipid disorders. It is also reported as a biomarker for cancer screening [101]. These evidential reports demand specific knowledge about the concentration of isoprene and acetone for the reliable diagnosis of the diseases. Since isoprene and acetone have a high dynamic range over the circadian cycle, therefore, it is important to know their concentration level, at the time of sample collection. This information is essential to upgrade the SOP for the diagnosis of disease.

### 4.5. Longitudinal Study of Metabolite’s Stability

**Figure 5:**
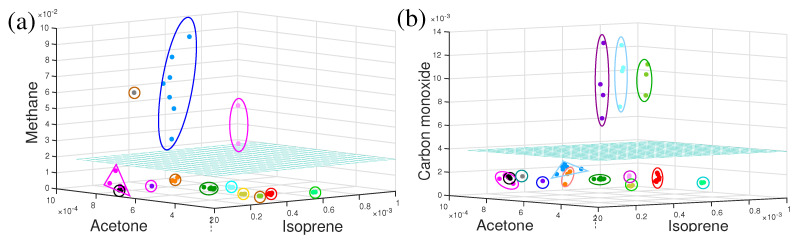
The three-dimensional component space representation of VOCs for healthy volunteers. Each enclosed cluster of points represents data from a single volunteer. The cyan colour horizontal plane separated (**a**) methanogenic and non-methanogenic volunteers and (**b**) smoker and non-smoker volunteers. (Ref. [17]).

To date, discussed dynamics of metabolites are for a short time period, extended from hours to a single day only. What would be the behaviour of metabolites over a longer time period such as months or even years? In a recent investigation, several healthy volunteers took part in a longitudinal breath study by infrared spectroscopy. Several VOCs were monitored over a time period of eighteen months and observations were plotted in three-dimensional VOCs space in Figure 5a,b. For each individual, measured points in three-dimensional component space were enclosed by a circle, oval, or triangle. For eleven out of fourteen individuals, measured points were enclosed in a compact space and clearly separated from each other (see Figure 5a). This means, the individual’s breath content remained stable over the period of eighteen months and the overall recognition score of the individual was 100%, meaning the probability of unambiguously linking the non-assigned experimental point to one or another individual. For three individuals, the size of the enclosures was relatively large in methane–acetone–isoprene space (Figure 5a) and they were well apart from the other eleven individuals. It was observed that these three individuals shifted far away from other only in the methane axis. The individual points were also elongated in the methane axis. These three individuals were high methane emitters and their methane concentration varies over time [102]. Measured points for these three individuals in CO–acetone–isoprene (Figure 5b) space were compact in a small space; however, three smokers were separated out, who were compact in the previous case.

**Figure 6:**
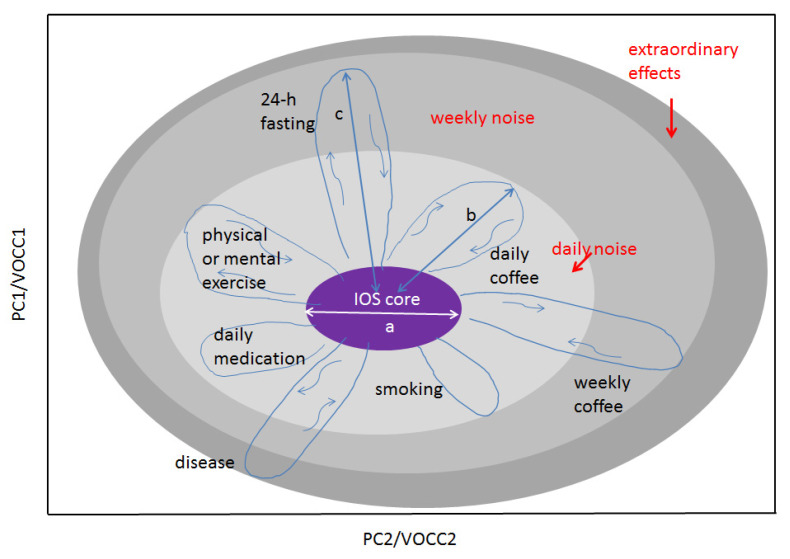
An illustration of the IOS concept for an individual. Any physiological parameters of the body can be presented on this graph. The space of representation can be blind (PC, canonical analysis) or show measurable variables such as VOC concentrations (VOCC) as its axes. Shown: the light grey area represents several daily factors affecting the IOS core and increasing thus the measurable IOS size; the medium grey area, factors affecting IOS on a weekly or monthly scale such as fasting or coffee intake for rare coffee drinkers; the dark grey area, extraordinary effects such as strong stress or disease. There are two main scales making the concept quantifiable: the core size a and the strength of the effect b, c, etc. In the case of VOCC representation, scale parameters a and b are reduced to n and δn. The concept can be extended to many individuals. In this case, two other scales should be used: a and l, where l is the distance between the IOS cores. The higher the space dimensionality, the more cross sections can be found where any two persons will have l > a. (Ref. [17]).

In the long-term monitoring of metabolites for individual healthy persons, it was observed that, for each individual, a set of metabolites in breath samples remains reproducible at least for 18 months. This unique behaviour of an individual’s breath component was conceptualized as “island of stability” (IOS) in a multidimensional metabolite concentration space. The concept is depicted in Figure 6. The IOS approach allows us to represent any physiological data of an individual as well as the effects affecting their variations, both in a quantifiable way. In the IOS representation, physiological parameters for an individual reduced to a multidimensional metabolite space as $({\bar{n}}_{1}\pm \delta n_{1},\cdots ,{\bar{n}}_{i}\pm \delta n_{i}),$ with $\delta n_{i}$ as concentration variations of *i*th metabolites during the longitudinal study. This set is called an IOS. In mathematical representation, a set of averaged values $\left({\bar{n}}_{1},\cdots ,{\bar{n}}_{i}\right)$ represents the IOS core of the individual’s data of the highest precision. In normal physiological conditions, the IOS of an individual represents a dressed state (or the noise-affected state containing all contributions to $\delta n_{i}$), marked in grey in Figure 6. Different sizes of grey areas represent different effects that influence the concentration of metabolites. When these effects are released, the concentration of metabolites returns to the IOS. Nonetheless, for disease cases, it takes a long path to return to IOS and in the case of chronic disease, the IOS shift does not return to the core.

**Figure 7:**
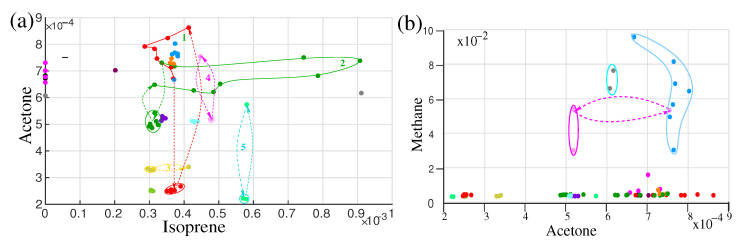
Two-dimensional illustration of different effects affecting the IOS on isoprene-acetone (**a**) and acetone-methane (**b**) plots. The same colours in the plots correspond to the same volunteers. Dashed lines are used to visualize the trajectory of the concentration variation of VOCs for different effects. The red colour plot represents the fasting effect and the green trajectory represents circadian variation. (Ref. [17]).

A disease is a change in the equilibrium state of the body, which may affect the metabolism of the body [103]. Due to the change in metabolism, it is expected that the characteristics of the contents of the biofluids (metabolites) also change. Having sensitive instruments for analysing the constitute and/or concentrations of the maximum amount of metabolites, it is possible to follow the body state and more importantly, one can try to recognize the disease at its early (asymptomatic) stage [17]. During the 18 months of study, some of the volunteers suffer from the common cold, pollen allergy, and viral and bacterial infections. The concentration of breath VOCs in the recovery time was monitored. The evolution path of the metabolites was drawn in Figure 7a,b. Although the monitoring was not systematic, many cases of intermediate states and return to the steady-state concentration of the metabolites after recovery indicate that it is possible to monitor the progression of the disease from its asymptomatic state. A discussion on diseases and potential biomarkers is presented in the following sections.

## 5. Diagnosis and Potential Biomarkers

It is an established fact that metabolites carry unique chemical information specific to the cellular processes of human beings. By characterizing metabolites, chemical processes in the cellular constitution can be revealed. In a steady health condition, constituent metabolites in biofluids are in equilibrium. In case of any abnormalities in cells, the reaction rate of the biochemical processes may change. As a result, constituent metabolites in biofluids deviate from their equilibrium state. Revealing the characteristic change of metabolites it may possible to identify the cells which are under an abnormal situation. This is the key point of a diagnosis of disease by analysing metabolites and thus a new branch of science has evolved, called metabolomics [104,105]. Metabolites related to the specific disease are called biomarkers. In pathology, chemical analysis of urine and blood are routine procedures for diagnosis and monitoring many diseases such as diabetes, Alzheimer’s, cardiovascular disease, prostate cancer, etc. Still, it does not consider an independent tool for diagnosis but rather considered as a supportive diagnostic tool. Significant work is going on to find out disease-specific reliable metabolites [16,106,107,108,109,110,111], more specifically which is called as a biomarker. A few disease-specific biomarkers are discussed below.

## 6. Disease Specific Biomarkers

For the diagnosis of a disease, it is necessary to follow the IOS for a person under investigation. Any deviation in the concentration of metabolites from IOS indicates an abnormality of the body which may relate to a disease [17]. To find out specific biomarkers for a particular disease, a common practice is to compare the biofluid of the diseased person with the healthy person. In the case of infrared spectroscopy, spectral features in the infrared spectra of biofluids may differ in one or many places for the healthy and disease cases. Those spectral regions are chosen for further analysis. To identify the biomarker, known molecular spectra are fitted with the identified spectral features in breath by least square fitting. The best fitted molecule is the possible biomarker for the disease. Finally, a statistical analysis is performed to find out the maximum number of variables possible among healthy and diseased groups.

### 6.1. Diabetes

**Figure 8:**
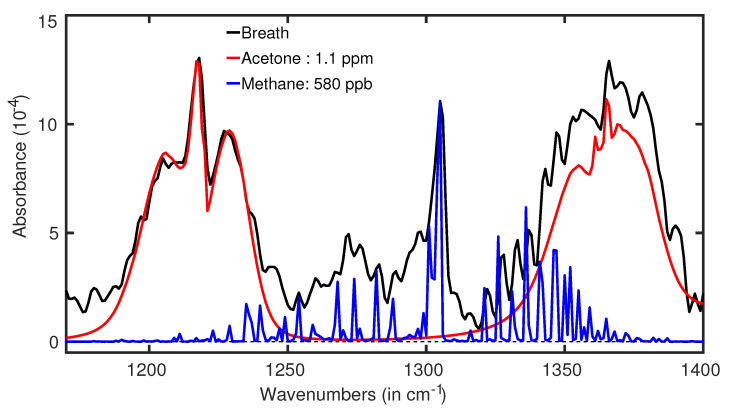
Acetone spectra in a real breath of a healthy volunteer (black line). The red plot is the fitting curve of acetone spectra. (Ref. [74]).

A metabolic disorder is a common heath issue among a large population in a modern society [5,112]. The metabolic disorder is commonly called diabetes. In general, a high blood sugar level is observed over a prolonged period of time in the case of diabetes [20]. It develops silently in the body and turns into a chronic disease. Over time, diabetes can cause serious health problems, such as heart disease, vision loss, kidney disease, etc. [21]. Biochemical analysis of blood is a routine procedure for clinical diagnosis and monitoring diabetes [113]. However, the invasive collection of the blood samples makes the diagnosis unpleasant for regular monitoring of blood sugar. Chemical analysis of saliva is a promising method for diagnosis and monitoring of sugar levels in the body [114]. The technique needs to be developed further before being accepted as a reliable clinical application. It is an established fact from ancient history that the body odour of diabetic patients smells sweet due to the presence of excess acetone in their biofluids, such as sweat, breath, etc. Recently many studies manifested that exhaled breath of diabetic patients contains considerably higher acetone than a healthy person [109,115,116,117,118]. Most of those breath measurements were performed by mass spectroscopic methods. As already mentioned, due to high cost, bulky size, and complicated sample preparation procedure, mass-spectroscopy is not yet a reliable technique for the diagnosis of diabetes. On the other hand, infrared spectroscopy has many advantages for detecting acetone in the biofluids [74,119,120]. In infrared spectra, acetone yields prominent distinguishable spectral features, which makes them easier to identify in gaseous biofluids. Quantum cascade laser has been used to analyse exhaled breath of patients with type 1 diabetes [120]. Acetone was identified, but due to the strong absorption line of water, the spectral feature of acetone looked quite noisy (see Figure 2 in Ref. [120]). In this experiment, water has been suppressed from the breath samples but the suppression was not sufficient. In a recent experiment, a thermal source-based FTIR spectrometer has been used to analyse exhaled breath samples from healthy volunteers. The sample was sent through a water condenser and a strong suppression of water vapour was achieved. The absorption spectra of breath in the fingerprint region of acetone are presented in Figure 8. Two broad peaks centred at 1217 cm${}^{-1}$ and 1365 cm${}^{-1}$ indicate the presence of acetone in the breath sample. The peak at 1217 cm${}^{-1}$ is practically noise-free (black line) and fitted quite well with the reference (PNNL [121]) fitting curve of acetone (red line). However, the right side spectral feature of breath spectra, centred at 1365 cm${}^{-1}$ is slightly elevated in amplitude than the corresponding acetone peak. Using a developed data analysis technique, it was confirmed that this peak is a result of the combined absorption of acetone, aldehyde, tetramethylurea, and some other unidentified molecules [122]. The acetone concentration was calculated by applying the least square fitting. The measured concentration of acetone for this particular volunteer is ∼1.1 ppm. The reported concentration of acetone for patients suffering from type 1 diabetes is in between 1.5 and 2.2 ppm [117,118]. Here the presented acetone fingerprints are for a single volunteer. Acetone concentration was also measured and monitored for a large number of healthy volunteers by infrared spectroscopy [17]. For all the cases, acetone concentrations are well below the range of type 1 diabetes cases. The measured acetone level of healthy volunteers is also supported by measuring acetone concentration by mass-spectroscopy [115,123]. Therefore, it can be concluded that infrared spectroscopy can be an alternative diagnostic method for diabetes. The precise measurements of the time evaluation of acetone concentration for the circadian cycle and after-fasting effects by infrared spectroscopy even strengthen the argument (see Section 4.3 and Section 4.4).

### 6.2. Antibiotic Treatment

Bacterial infection is a common cause of many diseases, e.g., cholera, tuberculosis, pneumonia, etc. Recently many studies have even found evidence that some cancers are initiated by bacteria [124,125,126,127]. Therefore, early detection of bacterial infection and monitoring its evolution for the diagnosis and treatment of many diseases is a necessary task. Current diagnostic methods to detect bacterial infection mostly rely on the culture of microorganisms from different biofluids. This approach is laborious and relatively time-consuming to obtain the result. In addition, culture-based diagnosis suffers from many pre-analytical limitations that may affect the performance of bacterial detection. For example, the inadequate volume of the collected biofluid, prior antibiotic exposure, contamination, and delays in laboratory processing are some of the main pre-analytical factors. In many cases, reliable identification of the infection and susceptibility testing may take a few days. Contamination is a frequent problem that may drive inappropriate antibiotic use, misdirect clinical diagnosis, and expose patients to unnecessary toxicities [128]. Many microbiological methods have been developed recently, which enhance the accuracy and faster detection of bacterial infection; however, the inherent problems of cultural approach are still there [129,130].

**Figure 9:**
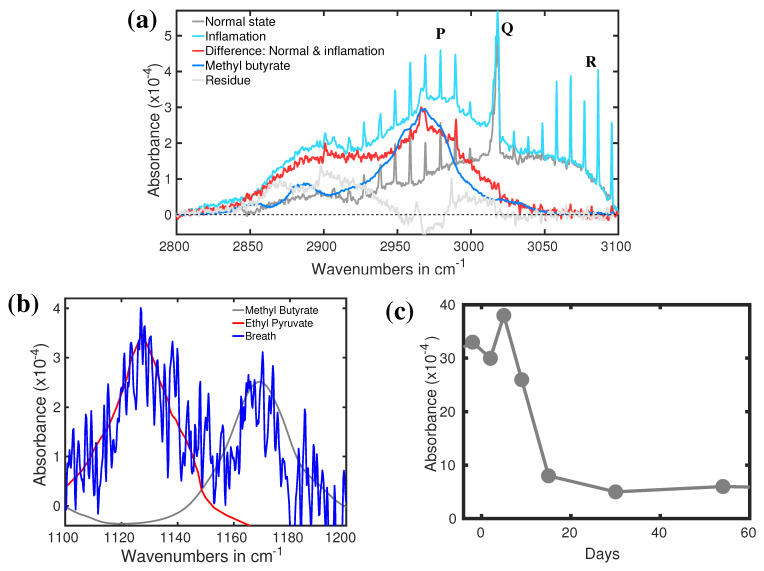
(**a**) Absorption spectra of breath when the person infected by bacteria. (Ref. [132]). (**b**) Absorption spectra of the breath of a healthy volunteer (grey) and in bacterial infection (cyan). The red curve is the spectral difference between the above two spectra. The blue plot represents the IR spectrum of methyl butyrate. (Ref. [122]). (**c**) The recovery dynamics of acute gastritis via QAC. (Ref. [132]).

Apart from the detection of bacterial infection, understanding the population dynamics of microbiota helps to develop an efficient antibiotic treatment. The current knowledge on deviations of human microbiota caused by antibiotic treatment is substandard [131]. To improve it, deviation of breath VOCs of a volunteer under treatment of quadruple antibiotic course (QAC) against *Helicobacter pylori* has been studied by infrared spectroscopy [132]. Two spectral regions were identified where the corresponding spectral structures strongly deviate during the antibiotic treatment [132]. Both spectral features along with the time trace of one of them are shown in Figure 9a–c. The spectral feature of methane at around 3000 cm${}^{-1}$ is strongly modified by some unknown spectral features. Using the digital subtraction method, a prominent spectral feature is revealed (see Figure 9b). This feature is well fitted with methyl butyrate absorption peak at around 2970 cm${}^{-1}$. A detailed of digital subtraction procedure is presented in a separate article [122]. Two other prominent spectral features are observed in Figure 9b. The spectra feature at around 1130 cm${}^{-1}$ is fitted well with the absorption peak of ethyl pyruvate and peak at around 1170 cm${}^{-1}$ is well fitted with methyl butyrate. Both the identified molecules generated by bacteria in the gut are involved in fundamental metabolic processes [107,133]. Therefore, both metabolites could be used for monitoring acute gastritis and anti-*Helicobacter pylori* treatment. The time trace of the absorption peak at 2970 cm${}^{-1}$ is plotted in Figure 9c. During infection by *Helicobacter pylori*, the concentration of methyl butyrate is quite high. After the antibiotic treatment, the concentration drops relatively slowly and takes more than 10 days to return to its normal concentration. To the best of my knowledge, this is the first demonstration of the dynamics of breath VOCs for acute gastritis affected by the quadruple antibiotics course carried out by infrared spectroscopy. Therefore, it can be concluded that infrared spectroscopy is capable of identifying possible biomarkers for bacterial infection. Of course, it needs to be clarified with some other bacteria also. In an ongoing project, many bacteria are identified by analysing bacterial headspace by infrared spectroscopy.

### 6.3. Cerebral Palsy

Cerebral palsy (CP) is a permanent disorder of the postural and musculoskeletal systems, caused by non-progressive damage to the brain in early childhood, shortly before, after, or during the birth [134,135,136]. It causes learning disabilities, behavioural problems, speech disorders, perception deficits, and seizure disorders [137,138]. Unfortunately little has been known about brain damage. Recently a mathematical model has been developed to understand the cerebral blood flow and occurrence of intracerebral haemorrhage in preterm infants. Based on this model, a machine learning model has been developed for identifying preterm infants who are at risk of cerebral haemorrhage [139]. This is a very important step toward understanding one of the causes of brain damage; however, its verification is far from reality, as the brain damage is understood much later than it actually occurs. Recently, in a pilot study, a postmortem brain biopsy was analysed using mass spectrometry and nuclear magnetic resonance spectroscopy [110]. Several potential biomarkers have been identified by both experimental techniques. The article reported the metabolomic profiling and biochemical pathways associated with CP. These findings definitely help to further investigation of the complex etiopathophysiology of CP.

**Figure 10:**
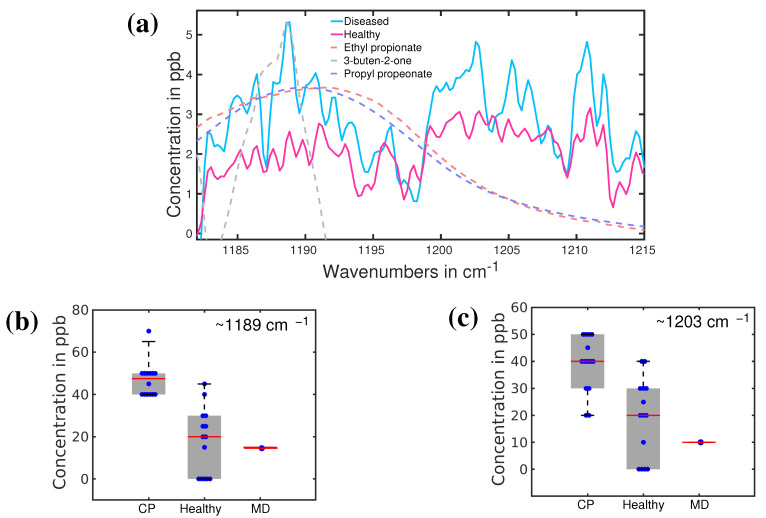
(**a**) The average absorption spectra of healthy (red) and CP (blue) volunteers. Dashed lines: fitting curves of three main VOC candidates; (**b**,**c**), plot boxes for healthy, CP, and a patient with muscular dystrophy (MD), with the corresponding median values and error bars. (Ref. [141]).

Recently another investigation was carried out to reveal possible biomarkers in the breath sample of a person with CP [140]. Infrared spectroscopy has been used to identify the VOCs associated with CP in exhaled breath. The infrared spectra of breath from 13 volunteers with CP were compared with 14 healthy volunteers of comparable ages. The average infrared spectra of CP and healthy cohorts are presented in Figure 10a. Infrared spectroscopy allowed us to identify two distinguishable spectral features for CP and healthy groups. These two spectral feature are observed around 1189 cm${}^{-1}$ and 1205 cm${}^{-1}$. The least-square fitting procedure was performed to find out the possible molecules associated with these two spectral features. Ethyl propionate, propyl propionate, and 3-buten-2-one seem to fit quite well with the spectral feature around 1189 cm${}^{-1}$. The other peak is not yet resolved. Statistical analysis was performed on the spectral data in the range 1185–1215 cm${}^{-1}$ using unsupervised principal component analysis (PCA analysis) and supervised Support Vector Machine (SVM) and Random Forest (RF) methods. The statistical results are presented in Figure 10b,c. More than 90% accuracy has been achieved in the identification of the two groups by using supervised analysis [141]. This is a significant result toward the development of the diagnosis of CP at an early stage. However, promising results demand additional studies, mostly focused on new specific biomarkers, larger statistics, and an extension of this investigation to newly born babies. All these three aspects are under investigation in an ongoing project.

### 6.4. Prostate Cancer

Cancer is one of the most vulnerable diseases and causes millions of deaths worldwide [1]. An uncontrollable cell growth can start in any part of the body and spread to other parts of the body. It might be prevented if affected cells are identified in their early disease state. Unfortunately, at an early stage, cancer remains asymptotic and difficult to realize. Symptoms only appear when cancer reaches its advanced stage and pushes the patient to a great risk of death. However, if the infected cells are identified, they can be completely cured, or at least the life span of the patient can be prolonged. Until now, invasive tissue collection and biopsy has been the only reliable diagnostic method for detecting cancer cells. This invasive process not only creates surgical complications but also creates psychological stress; therefore, it is used only with definite symptomatic cases. Unfortunately, it is already too late in many cases.

Many attempts have been taken to develop a non-invasive technique for the detection of cancer. Imaging-based tumour detection is one of the pioneering processes for cancer diagnosis [9,10]. However, due to technical reasons, small tumours cannot be detected at their initial stage by imaging techniques. Therefore, this technique is also applicable to the advanced stages of cancer detection. Alternatively, metabolic analysis of biofluids already demonstrated its power to detect many diseases, such as diabetes, cardiovascular disease, Alzheimer’s, etc. [11,12]. Therefore, it is logical to expect that analysis of metabolites in human biofluids may contribute to the diagnosis of cancer. Indeed, since the affected cells suffer from the uncontrolled high rate of cell division, their metabolism changes significantly, which is expected to reflect in biofluid.

Many studies have been performed to find out reliable biomarkers for different cancer types. Lung cancer is probably the most investigated cancer type for biomarker findings. The availability of different biofluids, which are directly related to the lung’s probably the reason behind the most investigated cancer types. Additionally, most of the biofluids for lung cancer investigation are collected through non-invasive, semi-invasive, or minimally-invasive processes. For example, exhaled breath can be collected fully non-invasively, and sputum can be collected non-invasively or semi-invasively [142,143]. Blood also consider as a biofluid for biomarker-based lung cancer detection. There has been significant work on biomarker search from exhaled breath. A large number of them have been carried out by mass-spectroscopy methods. Many biomarkers have been reported [26,144,145,146,147]; however, agreement among different researchers is not convincing. In spite of the poor agreement, biomarker-based lung cancer detection can be a potential non-invasive diagnostic for lung cancer. However, further improvement of the techniques and tests of a larger number of data sets is necessary to find out reliable VOCs related to lung cancer. Very few studies have been performed to reveal biomarkers for colon [148], bladder [149], breast [111], prostate [106], and other cancers. Recent work on prostate cancer detection by infrared spectroscopy is presented in the following section.

**Figure 11:**
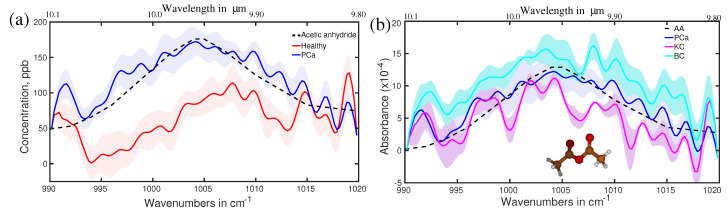
(**a**) Average absorption spectra of the healthy (red) and prostate cancer (blue) volunteers. The shaded plots are from each individual in respective sample groups. Dashed line: fitting curves of acetic anhydride, identified as biomarker. (**b**) Average absorption spectra of the prostate cancer (blue), bladder cancer (cyan), and kidney cancer (violate) patients. The molecular structure of acetic anhydride is presented as a ball-and-stick model. (Ref. [158]).

Prostate cancer may be the second most investigated cancer type for biomarkers search. In this regard, urine has been mostly investigated as biofluid as it is closely related to the prostate. Large metabolites in the liquid phase, as well as small metabolites (VOCs) in the gas phase from urine, are under investigation [106,150,151,152]. Prostate-specific antigen (PSA) test from the blood serum is one of the routine screening procedures for the detection of prostate cancer; however, the sensitivity and the specificity are significantly lower than the desired accuracy for cancer diagnosis [153,154]. Breath has been also considered a potential source of biomarkers for prostate cancer [155,156]. A little has been conducted to find out breath VOCs related to prostate cancer. Recently, an infrared spectroscopy-based VOCs analysis for prostate cancer demonstrated promising results. In this study, breath samples from a small cohort of 28 prostate cancer patients were analysed and compared with 19 healthy volunteers [157]. In addition, breath samples of eight kidney cancer and eight bladder cancer cases were also investigated to find out whether the infrared signatures of all these three cancers are different or not. Eight spectral regions were found where spectral signatures for different cohorts are distinguishable. One of the spectral signatures, centred around 1005 cm${}^{-1}$, is shown in Figure 11a for the healthy and prostate cancer cohorts. The average spectra of the healthy cohort are shown with a solid red line. A shaded red region depicts the spectral variation of each individual. Similarly, prostate cancer cohorts are shown with blue colour. Two cohorts are fully separated even without overlapping among their individuals. This allows for distinguishing prostate cancer cohorts from healthy cohorts with over 95% accuracy [158]. The three types of cancer under investigation are also distinguishable from each other; however, their separations are not prominent like in the previous case (see Figure 11b). The molecule associated with this spectral feature was identified by least-square fitting with reference molecular spectra from a commercial database. For this case, acetic anhydride is identified as one of the potential biomarkers for prostate cancer diagnosis. The identification was also verified by quantum chemistry calculations (see supplementary of reference [158]). In this study, seven more spectral features are identified for which healthy and prostate cancer cohorts are distinguished with accuracy over 90%. A detail of the spectral position, associated molecule, and calculated accuracy are presented in Table 1.

**Figure 12:**
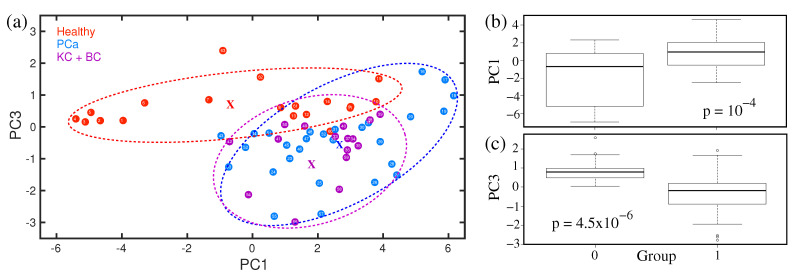
(**a**) Statistical analysis using PCA method. Ellipses are shown for visualization, with the corresponding centres marked by “x”. (**b**,**c**) The box plots of statistical results. (Ref. [158]).

Statistical analysis is performed in case of visible spectral differences among the different sets of infrared spectra of breath to determine the accuracy of the diagnosis. Selected statistical results are presented in Figure 12a–c. Supervised as well as unsupervised statistical analyses were performed. The PCA analysis of spectral feature around 1005 cm${}^{-1}$ is presented in Figure 12a. The principle component values for different cohorts are clustered in PC1 vs. PC3 space. For a better understanding, the clusters are enclosed by ellipses. It was revealed that healthy and prostate cancer groups are well separated. The cloud for bladder and kidney cases is more compact; however, overlapped with the prostate cancer cloud. It is not clear yet, whether urogenital cancers are indistinguishable with respect to this particular volatile metabolite or insufficient sample size, especially for bladder and kidney cancers made the analysis poor. A larger sample size is necessary to confirm the above statement, which is an ongoing project. The statistical data are also presented as box plots (see Figure 12b,c). The box plots demonstrate a good separation between healthy and prostate cancer groups. A figure of merit is determined by the *p*-value. In the case of *p* > 0.05, the data of the corresponding samples are dependent. The very low *p*-value for the spectral feature at 1005 cm${}^{-1}$ indicates that data from healthy and prostate cancer groups are independent. Similar results have been achieved for the other seven spectral regions where visible spectral differences are observed. The results are summarized in Table 1. 

## 7. Applicability of Infrared Technique for VOC Detection

It has been already demonstrated that infrared spectroscopy has the potential to identify disease-specific volatile biomarkers with high accuracy. Since it uses fundamental molecular vibrations as a probe to identify the molecules, the identification is straightforward and accurate. A minimal sample preparation effort makes the infrared detection techniques much faster than mass-spectroscopic methods for VOC detection [158]. In the current state, a typical measurement takes about twenty minutes including sample preparation, measurement, and cleaning of the system to prepare for the next measurement. However, regarding the number of molecules and detection sensitivity, infrared spectroscopy is far behind mass-spectroscopic techniques. Mass-spectroscopic techniques already identified hundreds of metabolites with a sensitivity of 100 ppt [26,30]. Of course, it needs to be noted that mass-spectroscopy for VOC detection has been developing for decades, whereas the infrared technique for VOC identification in biofluids is still in its infancy. Extensive research needs to be undertaken for spectroscopic technology development as well as VOC identification processes. For example, laser-based spectroscopy definitely increases the sensitivity of the detection down to the ppt level [159,160], which is comparable to the detection limit by mass-spectrometry. Regarding the device cost and size, infrared spectrometers are more favourable than mass-spectrometers. In fact, to identify specific molecules, extremely small-size single-frequency lasers can be made. Such laser systems are used in environmental, forensic, and food industries for the detection of volatile molecules. For clinical application, extensive research needs to be completed both in technology development as well as diagnostic development [158,161,162,163]. In the current state, it requires a molecular spectroscopic expert for the analysis of the data; however, when a disease-specific biomarker is confirmed, this diagnostic can be carried out by any clinician. In the same race, e-nose technology is also promising, especially for its size and cost; however, a careful investigation is necessary before it can be used as a clinical diagnostic method [164,165].

## 8. Conclusions

This review article presented the state-of-the-art applicability of infrared spectroscopy for volatile metabolites analysis in gaseous biofluids. It is an established fact that volatile metabolites present in biofluids carry information about the body’s state. As a consequence, abnormality/disease initiated at any part of the body is reflected in the composition of biofluids. By having sensitive instruments for analysing the change of equilibrium in metabolic composition, one can try to recognize the disease in its early (asymptomatic) state. Among many developing experimental techniques, infrared spectroscopy demonstrated very promising results. Infrared spectroscopy is a well-established experimental tool for molecular identification. However, due to a large amount of water contained, its applicability for biofluid analysis was limited until recent years. The development of the high-duty cycle water suppression technique for gaseous biological samples opens a new window for the applicability of infrared spectroscopy in biofluids. A detail of the experimental technique and data analysis are presented in this review. It has been shown how the VOC compositions in breath change with the circadian rhythm and external factors such as food, drink, physical exercise, etc. In spite of short-term changes, in general, VOC compositions remain stable over months and years unless the patient is suffering from a disease. This substantial evidence allowed us to establish the concept of the “Island of stability (IOS)”. Any deviation from IOS is considered an abnormality of the body. Infrared spectroscopy is capable to monitor IOS very precisely. Using this concept, the infection of Helicobacter pylori and its antibiotic treatment were monitored by infrared spectroscopy. This study identified the possible biomarker for bacterial infection caused by Helicobacter pylori. This experimental technique is also used to identify biomarkers for cerebral palsy and prostate cancer. Several possible biomarkers are identified for both disease cases. This technique also shows very high sensitivity and specificity (>90%) to distinguish between different diseases and healthy groups. All these pieces of evidence manifest that infrared spectroscopy holds a lot of promise as a future non-invasive diagnostic tool. However, significant developments need to be completed in terms of technology as well as data analysis before it will be accepted as a diagnostic tool. Many studies are being conducted in both directions to develop non-invasive diagnosis tools. 

## Figures and Tables

**Table 1:** Identified spectral regions (SR), corresponding molecules, and results from different statistical analysis methods for healthy and three urogenital cancers. (Ref. [158]).

The Center of SR, cm${}^{-1}$	Identified Metabolite	Metabolite Molecular Mass, *amu*	Concentration for Healthy in ppb, the Concentration Ratio of Cancer to Healthy	*p*-Values for PC1/PC2/PC3/ PC4 Analysis, Healthy vs. Cancer	Variance of PC1/PC2/PC3/ PC4 in %	9-Fold Validation: Accuracy/ Sensitivity/ Specificity/Error (SD), Healthy vs. PCa	9-Fold Validation: Accuracy/ Sensitivity/ Specificity/Error (SD), Healthy vs. PCa + BC + KC	7-Fold Validation: Accuracy/ Sensitivity/ Specificity/Error (SD), Healthy vs. BC + KC
1005	Acetic anhydride	102.09	83, 2.1	$1\times 10^{-4}$/$3\times 10^{-1}$ $5\times 10^{-6}$/$4\times 10^{-1}$	45/9/7/6	0.98/0.99/0.97/0.02	0.98/0.99/0.97/0.02	0.95/0.94/0.96/0.04
1190	Propyl propionate	116.16	87, 1.4	$4\times 10^{-1}$/$6\times 10^{-4}$ $3\times 10^{-2}$/$6\times 10^{-1}$	37/18/10/7	0.97/0.97/0.97/0.02	0.97/0.97/0.97/0.02	0.97/0.98/0.96/0.03
1203	Ethyl vinyl ketone	84.12	130, 0.8	$4\times 10^{-1}$/$6\times 10^{-4}$ $3\times 10^{-2}$/$6\times 10^{-1}$	37/18/10/7	0.97/0.97/0.97/0.02	0.97/0.97/0.97/0.02	0.97/0.98/0.96/0.03
530	Acetalde- hyde	44.05	690, 1.4	$3\times 10^{-4}$/$4\times 10^{-2}$ $6\times 10^{-2}$/$6\times 10^{-1}$	76/6/2/1	0.83/0.95/0.66/0.02	0.83/0.95/0.66/0.02	0.94/1.0/0.85/0.02
1050	Carbon dioxide	44.01	$4\times 10^{7}$, 0.8	$3\times 10^{-1}$/$6\times 10^{-2}$ $1\times 10^{-2}$/$1\times 10^{-2}$	88/6/2/1	0.92/0.97/0.86/0.02	0.92/0.97/0.86/0.02	0.87/0.87/0.87/0.02
2170	Carbon monoxide	28.01	$2\times 10^{3}$, 1.1	$9\times 10^{-1}$/$1\times 10^{-1}$ $1\times 10^{-2}$/$4\times 10^{-2}$	88/6/2/1	0.79/0.88/0.68/0.07	0.79/0.88/0.68/0.04	0.81/0.68/0.92/0.03
1130	Ethyl pyruvate	116.11	183, 0.8	$5\times 10^{-1}$/$5\times 10^{-1}$ $9\times 10^{-1}$/$1\times 10^{-1}$	26/17/9/9	0.99/0.99/0.99/0.02	0.99/0.99/0.99/0.02	0.95/0.93/0.96/0.04
1170	Methyl butyrate	102.13	100, 1.4	$3\times 10^{-1}$/$9\times 10^{-2}$ $2\times 10^{-1}$/$6\times 10^{-2}$	28/17/10/9	0.98/0.98/0.98/0.02	0.98/0.99/0.98/0.02	0.94/0.93/0.96/0.04

## Data Availability

Not applicable.

## References

[1] Sung H., Ferlay J., Siegel R.L., Laversanne M., Soerjomataram I., Jemal A., Bray F. (2021). Global Cancer Statistics 2020: GLOBOCAN Estimates of Incidence and Mortality Worldwide for 36 Cancers in 185 Countries. CA Cancer J. Clin..

[2] Virani S.S., Alonso A., Aparicio H.J., Benjamin E.J., Bittencourt M.S., Callaway C.W., Carson A.P., Chamberlain A.M., Cheng S., Delling F.N. (2021). Heart Disease and Stroke Statistics—2021 Update. Circulation.

[3] Tsao C.W., Aday A.W., Almarzooq Z.I., Alonso A., Beaton A.Z., Bittencourt M.S., Boehme A.K., Buxton A.E., Carson A.P., Commodore-Mensah Y. (2022). Heart Disease and Stroke Statistics—2022 Update: A Report From the American Heart Association. Circulation.

[4] Barnes P.J., Burney P.G.J., Silverman E.K., Celli B.R., Vestbo J., Wedzicha J.A., Wouters E.F.M. (2015). Chronic obstructive pulmonary disease. Nat. Rev. Dis. Prim..

[5] Kitabchi A.E., Umpierrez G.E., Miles J.M., Fisher J.N. (2009). Hyperglycemic Crises in Adult Patients with Diabetes. Diabetes Care.

[6] Knopman D.S., Amieva H., Petersen R.C., Chételat G., Holtzman D.M., Hyman B.T., Nixon R.A., Jones D.T. (2021). Alzheimer disease. Nat. Rev. Dis. Prim..

[7] Singer S. (2017). Psychosocial Impact of Cancer. Recent Results in Cancer Research.

[8] Niedzwiedz C.L., Knifton L., Robb K.A., Katikireddi S.V., Smith D.J. (2019). Depression and anxiety among people living with and beyond cancer: A growing clinical and research priority. BMC Cancer.

[9] Bi W.L., Hosny A., Schabath M.B., Giger M.L., Birkbak N.J., Mehrtash A., Allison T., Arnaout O., Abbosh C., Dunn I.F. (2019). Artificial intelligence in cancer imaging: Clinical challenges and applications. CA Cancer J. Clin..

[10] Meissner V.H., Rauscher I., Schwamborn K., Neumann J., Miller G., Weber W., Gschwend J.E., Eiber M., Heck M.M. (2022). Radical Prostatectomy Without Prior Biopsy Following Multiparametric Magnetic Resonance Imaging and Prostate-specific Membrane Antigen Positron Emission Tomography. Eur. Urol..

[11] DeBerardinis R.J., Keshari K.R. (2022). Metabolic analysis as a driver for discovery, diagnosis, and therapy. Cell.

[12] Mi K., Jiang Y., Chen J., Lv D., Qian Z., Sun H., Shang D. (2020). Construction and Analysis of Human Diseases and Metabolites Network. Front. Bioeng. Biotechnol..

[13] Metzler D.E. (2003). Biochemistry: The Chemical Reactions of Living Cells.

[14] Ahern K. (2019). Biochemistry and Molecular Biology: How Life Works.

[15] Berg J.M., Tymoczko J.L., Stryer L. (2002). Biochemistry.

[16] Sussulini A. (2017). Metabolomics: From Fundamentals to Clinical Applications.

[17] Maiti K.S., Lewton M., Fill E., Apolonski A. (2019). Human beings as islands of stability: Monitoring body states using breath profiles. Sci. Rep..

[18] Rowan D.D. (2011). Volatile Metabolites. Metabolites.

[19] Yi L.S., Chin T.L., Mohamad M.S., Deris S., Subair S., Ibrahim Z. (2015). A Review on Metabolic Pathway Analysis in Biological Production. Mini-Rev. Org. Chem..

[20] Shepherd P.R., Kahn B.B. (1999). Glucose Transporters and Insulin Action—Implications for Insulin Resistance and Diabetes Mellitus. N. Engl. J. Med..

[21] Emerging Risk Factors Collaboration (2010). Diabetes mellitus, fasting blood glucose concentration, and risk of vascular disease: A collaborative meta-analysis of 102 prospective studies. Lancet.

[22] Rosario D.J., Lane J.A., Metcalfe C., Donovan J.L., Doble A., Goodwin L., Davis M., Catto J.W.F., Avery K., Neal D.E. (2012). Short term outcomes of prostate biopsy in men tested for cancer by prostate specific antigen: Prospective evaluation within ProtecT study. BMJ.

[23] Pham Y.L., Beauchamp J. (2021). Breath Biomarkers in Diagnostic Applications. Molecules.

[24] Khoubnasabjafari M., Mogaddam M.R.A., Rahimpour E., Soleymani J., Saei A.A., Jouyban A. (2022). Breathomics: Review of Sample Collection and Analysis, Data Modeling and Clinical Applications. Crit. Rev. Anal. Chem..

[25] Maiti K.S., Roy S., Lampe R., Apolonski A. (2021). Detection of Disease-Specific Volatile Organic Compounds Using Infrared Spectroscopy. Eng. Proc..

[26] Ferraris V.A. (2016). What do dogs, ancient Romans, Linus Pauling, and mass spectrometry have in common? Early lung cancer and exhaled breath. J. Thorac. Cardiovasc. Surg..

[27] Williams H., Pembroke A. (1989). Sniffer dogs in the melanoma clinic?. Lancet.

[28] Church J., Williams H. (2001). Another sniffer dog for the clinic?. Lancet.

[29] Pauling L., Robinson A.B., Teranishi R., Cary P. (1971). Quantitative Analysis of Urine Vapor and Breath by Gas-Liquid Partition Chromatography. Proc. Natl. Acad. Sci. USA.

[30] de Lacy Costello B., Amann A., Al-Kateb H., Flynn C., Filipiak W., Khalid T., Osborne D., Ratcliffe N.M. (2014). A review of the volatiles from the healthy human body. J. Breath Res..

[31] Lamote K., Van Cleemput J., Nackaerts K., Vandermeersch L., Van Langenhove H., van Meerbeeck J.P. (2016). Breath analysis by gas chromatography-mass spectrometry can be used to screen for pleural mesothelioma. Eur. Respir. J..

[32] Lanucara F., Holman S.W., Gray C.J., Eyers C.E. (2014). The power of ion mobility-mass spectrometry for structural characterization and the study of conformational dynamics. Nat. Chem..

[33] Hagemann L.T., Repp S., Mizaikoff B. (2019). Hybrid Analytical Platform Based on Field-Asymmetric Ion Mobility Spectrometry, Infrared Sensing, and Luminescence-Based Oxygen Sensing for Exhaled Breath Analysis. Sensors.

[34] Ellis A.M., Mayhew C.A. (2014). Proton Transfer Reaction Mass Spectrometry: Principles and Applications.

[35] Smith D., Španěl P., Enderby B., Lenney W., Turner C., Davies S.J. (2010). Isoprene levels in the exhaled breath of 200 healthy pupils within the age range 7–18 years studied using SIFT-MS. J. Breath Res..

[36] Li C., Chu S., Tan S., Yin X., Jiang Y., Dai X., Gong X., Fang X., Tian D. (2021). Towards Higher Sensitivity of Mass Spectrometry: A Perspective From the Mass Analyzers. Front. Chem..

[37] Hanna G.B., Boshier P.R., Markar S.R., Romano A. (2019). Accuracy and Methodologic Challenges of Volatile Organic Compound–Based Exhaled Breath Tests for Cancer Diagnosis. JAMA Oncol..

[38] Lourenço C., Turner C. (2014). Breath Analysis in Disease Diagnosis: Methodological Considerations and Applications. Metabolites.

[39] Karakaya D., Ulucan O., Turkan M. (2020). Electronic Nose and Its Applications: A Survey. Int. J. Autom. Comput..

[40] Ye Z., Liu Y., Li Q. (2021). Recent Progress in Smart Electronic Nose Technologies Enabled with Machine Learning Methods. Sensors.

[41] Nakhleh M.K., Amal H., Jeries R., Broza Y.Y., Aboud M., Gharra A., Ivgi H., Khatib S., Badarneh S., Har-Shai L. (2017). Diagnosis and Classification of 17 Diseases from 1404 Subjects via Pattern Analysis of Exhaled Molecules. ACS Nano.

[42] Natale C.D., Paolesse R., Martinelli E., Capuano R. (2014). Solid-state gas sensors for breath analysis: A review. Anal. Chim. Acta.

[43] Patimisco P., Sampaolo A., Zheng H., Dong L., Tittel F.K., Spagnolo V. (2016). Quartz–enhanced photoacoustic spectrophones exploiting custom tuning forks: A review. Adv. Phys. X.

[44] Sampaolo A., Patimisco P., Giglio M., Zifarelli A., Wu H., Dong L., Spagnolo V. (2022). Quartz-enhanced photoacoustic spectroscopy for multi-gas detection: A review. Anal. Chim. Acta.

[45] Wilson E.B.J., Decius J.C., Cross P.C. (1955). Molecular Vibrations: The Theory of Infrared and Raman Vibrational Spectra.

[46] Maiti K.S. (2015). Broadband two dimensional infrared spectroscopy of cyclic amide 2-Pyrrolidinone. Phys. Chem. Chem. Phys..

[47] Fayer M.D. (2013). (Ed.) Ultrafast Infrared Vibrational Spectroscopy.

[48] Maiti K.S. (2020). Ultrafast vibrational coupling between C–H and C=O band of cyclic amide 2-Pyrrolidinone revealed by 2DIR spectroscopy. Spectrochim. Acta Part Mol. Biomol. Spectrosc..

[49] Maiti K.S. (2021). Two-dimensional Infrared Spectroscopy Reveals Better Insights of Structure and Dynamics of Protein. Molecules.

[50] Maiti K.S. (2007). High Level Ab Initio Potential Energy Surfaces and Vibrational Spectroscopy. Ph.D. Thesis.

[51] Maiti K.S., Samsonyuk A., Scheurer C., Steinel T. (2012). Hydrogen bonding characteristics of 2-pyrrolidinone: A joint experimental and theoretical study. Phys. Chem. Chem. Phys..

[52] Arrondo J.L.R., Muga A., Castresana J., Goñi F.M. (1993). Quantitative studies of the structure of proteins in solution by fourier-transform infrared spectroscopy. Prog. Biophys. Mol. Biol..

[53] Marco J., Orza J., Abboud J.L. (1994). Fourier transform infrared study of gas phase H-bonding: Absorptivities and formation equilibrium constants of fluoroalcohol complexes. Vib. Spectrosc..

[54] Roy S., Maiti K.S. (2018). Structural sensitivity of CH vibrational band in methyl benzoate. Spectrochim. Acta Mol. Biomol. Spectrosc..

[55] Meganathan C., Sebastian S., Kurt M., Lee K.W., Sundaraganesan N. (2010). Molecular structure, spectroscopic (FTIR, FTIR gas phase, FT-Raman) first-order hyperpolarizability and HOMO–LUMO analysis of 4-methoxy-2-methyl benzoic acid. J. Raman Spectrosc..

[56] Heise H.M., Mink J., Keresztury G., Kellner R. (1997). Medical Applications of Infrared Spectroscopy. Proceedings of the Progress in Fourier Transform Spectroscopy.

[57] Maiti K.S. (2015). Vibrational spectroscopy of Methyl benzoate. Phys. Chem. Chem. Phys..

[58] Maiti K.S., Scheurer C. (2013). Basis Set Extrapolation for the High Resolution Spectroscopy. J. Chem. Chem. Eng..

[59] Maiti K.S. (2018). Ultrafast N–H vibrational dynamics of hydrogen-bonded cyclic amide reveal by 2DIR spectroscopy. Chem. Phys..

[60] Probst D., Reymond J. (2018). A probabilistic molecular fingerprint for big data settings. J. Cheminform..

[61] Bakker J.M., Aleese L.M., Meijer G., von Helden G. (2003). Fingerprint IR Spectroscopy to Probe Amino Acid Conformations in the Gas Phase. Phys. Rev. Lett..

[62] Takamura A., Watanabe K., Akutsu T., Ozawa T. (2018). Soft and Robust Identification of Body Fluid Using Fourier Transform Infrared Spectroscopy and Chemometric Strategies for Forensic Analysis. Sci. Rep..

[63] Yu M.C., Rich P., Foreman L., Smith J., Yu M.S., Tanna A., Dibbur V., Unwin R., Tam F.W.K. (2017). Label Free Detection of Sensitive Mid-Infrared Biomarkers of Glomerulonephritis in Urine Using Fourier Transform Infrared Spectroscopy. Sci. Rep..

[64] Baker M.J., Trevisan J., Bassan P., Bhargava R., Butler H.J., Dorling K.M., Fielden P.R., Fogarty S.W., Fullwood N.J., Heys K.A. (2014). Using Fourier transform IR spectroscopy to analyze biological materials. Nat. Protoc..

[65] Huber M., Kepesidis K.V., Voronina L., Fleischmann F., Fill E., Hermann J., Koch I., Milger-Kneidinger K., Kolben T., Schulz G.B. (2021). Infrared molecular fingerprinting of blood-based liquid biopsies for the detection of cancer. eLife.

[66] Guang P., Huang W., Guo L., Yang X., Huang F., Yang M., Wen W., Li L. (2020). Blood-based FTIR-ATR spectroscopy coupled with extreme gradient boosting for the diagnosis of type 2 diabetes. Medicine.

[67] Silva L.G., Péres A.F.S., Freitas D.L.D., Morais C.L.M., Martin F.L., Crispim J.C.O., Lima K.M.G. (2020). ATR-FTIR spectroscopy in blood plasma combined with multivariate analysis to detect HIV infection in pregnant women. Sci. Rep..

[68] Martinez-Cuazitl A., Vazquez-Zapien G.J., Sanchez-Brito M., Limon-Pacheco J.H., Guerrero-Ruiz M., Garibay-Gonzalez F., Delgado-Macuil R.J., de Jesus M.G.G., Corona-Perezgrovas M.A., Pereyra-Talamantes A. (2021). ATR-FTIR spectrum analysis of saliva samples from COVID-19 positive patients. Sci. Rep..

[69] Caixeta D.C., Lima C., Xu Y., Guevara-Vega M., Espindola F.S., Goodacre R., Zezell D.M., Sabino-Silva R. (2023). Monitoring glucose levels in urine using FTIR spectroscopy combined with univariate and multivariate statistical methods. Spectrochim. Acta Part A Mol. Biomol. Spectrosc..

[70] Malek K., Wood B.R., Bambery K.R. (2013). FTIR Imaging of Tissues: Techniques and Methods of Analysis. Challenges and Advances in Computational Chemistry and Physics.

[71] Ali M.H.M., Rakib F., Al-Saad K., Al-Saady R., Goormaghtigh E. (2019). An Innovative Platform Merging Elemental Analysis and Ftir Imaging for Breast Tissue Analysis. Sci. Rep..

[72] Phillips M., Herrera J., Krishnan S., Zain M., Greenberg J., Cataneo R.N. (1999). Variation in volatile organic compounds in the breath of normal humans. J. Chromatogr. B Biomed. Sci. Appl..

[73] Zieliński J., Przybylski J. (2012). How much water is lost during breathing?. Pneumonol. Alergol. Pol..

[74] Maiti K.S., Lewton M., Fill E., Apolonski A. (2018). Sensitive spectroscopic breath analysis by water condensation. J. Breath Res..

[75] Apolonski A., Roy S., Lampe R., Maiti K.S. (2020). Molecular identification of bio-fluids in gas phase using infrared spectroscopy. Appl. Opt..

[76] Beauchamp J., Herbig J., Gutmann R., Hansel A. (2008). On the use of Tedlar bags for breath-gas sampling and analysis. J. Breath Res..

[77] Lawal O., Ahmed W.M., Nijsen T.M.E., Goodacre R., Fowler S.J. (2017). Exhaled breath analysis: A review of ‘breath-taking’ methods for off-line analysis. Metabolomics.

[78] Kang S., Thomas C.L.P. (2016). How long may a breath sample be stored for at −80 °C? A study of the stability of volatile organic compounds trapped onto a mixed Tenax: Carbograph trap adsorbent bed from exhaled breath. J. Breath Res..

[79] Apolonski A., Roy S., Lampe R., Maiti K.S. (2019). Application of Vibrational Spectroscopy in Biology and Medicine. Breath Analysis. Proceedings.

[80] Gelin M.F., Blokhin A.P., Ostrozhenkova E., Apolonski A., Maiti K.S. (2021). Theory helps experiment to reveal VOCs in human breath. Spectrochim. Acta Part A Mol. Biomol. Spectrosc..

[81] Holmes E., Wilson I.D., Nicholson J.K. (2008). Metabolic Phenotyping in Health and Disease. Cell.

[82] Assfalg M., Bertini I., Colangiuli D., Luchinat C., Schäfer H., Schütz B., Spraul M. (2008). Evidence of different metabolic phenotypes in humans. Proc. Natl. Acad. Sci. USA.

[83] Wallner-Liebmann S., Tenori L., Mazzoleni A., Dieber-Rotheneder M., Konrad M., Hofmann P., Luchinat C., Turano P., Zatloukal K. (2016). Individual Human Metabolic Phenotype Analyzed by 1H NMR of Saliva Samples. J. Proteome Res..

[84] Yousri N.A., Kastenmüller G., Gieger C., Shin S.Y., Erte I., Menni C., Peters A., Meisinger C., Mohney R.P., Illig T. (2014). Long term conservation of human metabolic phenotypes and link to heritability. Metabolomics.

[85] Ghini V., Saccenti E., Tenori L., Assfalg M., Luchinat C. (2015). Allostasis and Resilience of the Human Individual Metabolic Phenotype. J. Proteome Res..

[86] Martinez-Lozano Sinues P., Kohler M., Zenobi R. (2013). Human Breath Analysis May Support the Existence of Individual Metabolic Phenotypes. PLoS ONE.

[87] King J., Kupferthaler A., Unterkofler K., Koc H., Teschl S., Teschl G., Miekisch W., Schubert J., Hinterhuber H., Amann A. (2009). Isoprene and acetone concentration profiles during exercise on an ergometer. J. Breath Res..

[88] Lovallo W.R., Farag N.H., Vincent A.S., Thomas T.L., Wilson M.F. (2006). Cortisol responses to mental stress, exercise, and meals following caffeine intake in men and women. Pharmacol. Biochem. Behav..

[89] Kasapis C., Thompson P.D. (2005). The Effects of Physical Activity on Serum C-Reactive Protein and Inflammatory Markers: A Systematic Review. J. Am. Coll. Cardiol..

[90] Espersen G.T., Elbaek A., Ernst E., Toft E., Kaalund S., Jersild C., Grunnet N. (1990). Effect of physical exercise on cytokines and lymphocyte subpopulations in human peripheral blood. APMIS.

[91] Ciloglu F., Peker I., Pehlivan A., Karacabey K., İlhan N., Saygin O., Ozmerdivenli R. (2005). Exercise intensity and its effects on thyroid hormones. Neuroendocrinol. Lett..

[92] Raninen K.J., Lappi J.E., Mukkala M.L., Tuomainen T.P., Mykkänen H.M., Poutanen K.S., Raatikainen O.J. (2016). Fiber content of diet affects exhaled breath volatiles in fasting and postprandial state in a pilot crossover study. Nutr. Res..

[93] Zakhari S. (2006). Overview: How is alcohol metabolized by the body?. Alcohol Res. Health J. Natl. Inst. Alcohol Abus. Alcohol..

[94] Smith D., Španěl P., Davies S. (1999). Trace gases in breath of healthy volunteers when fasting and after a protein-calorie meal: A preliminary study. J. Appl. Physiol..

[95] Meyer B., Scholtz H., Schall R., Muller F., Hundt H., Maree J. (1995). The effect of fasting on total serum bilirubin concentrations. Br. J. Clin. Pharmacol..

[96] Landaw S.A., Callahan E.W., Schmid R. (1970). Catabolism of heme in vivo: Comparison of the simultaneous production of bilirubin and carbon monoxide. J. Clin. Investig..

[97] Jones A. (1987). Breath-Acetone Concentrations in Fasting Healthy Men: Response of Infrared Breath-Alcohol Analyzers. J. Anal. Toxicol..

[98] King J., Kupferthaler A., Frauscher B., Hackner H., Unterkofler K., Teschl G., Hinterhuber H., Amann A., Högl B. (2012). Measurement of endogenous acetone and isoprene in exhaled breath during sleep. Physiol. Meas..

[99] Gelmont D., Stein R.A., Mead J.F. (1981). Isoprene—The main hydrocarbon in human breath. Biochem. Biophys. Res. Commun..

[100] Sharkey T.D. (1996). Isoprene synthesis by plants and animals. Endeavour.

[101] Salerno-Kennedy R., Cashman K. (2005). Potential applications of breath isoprene as a biomarker in modern medicine: A concise overview. Wien Klin. Wochenschr..

[102] Polag D., Keppler F. (2018). Long-term monitoring of breath methane. Sci. Total Environ..

[103] Pauling L. (1968). Orthomolecular Psychiatry. Science.

[104] Thistlethwaite L.R., Li X., Burrage L.C., Riehle K., Hacia J.G., Braverman N., Wangler M.F., Miller M.J., Elsea S.H., Milosavljevic A. (2022). Clinical diagnosis of metabolic disorders using untargeted metabolomic profiling and disease-specific networks learned from profiling data. Sci. Rep..

[105] Gowda G.N., Zhang S., Gu H., Asiago V., Shanaiah N., Raftery D. (2008). Metabolomics-based methods for early disease diagnostics. Expert Rev. Mol. Diagn..

[106] Lima A.R., Pinto J., Azevedo A.I., Barros-Silva D., Jerónimo C., Henrique R., de Lourdes Bastos M., Guedes de Pinho P., Carvalho M. (2019). Identification of a biomarker panel for improvement of prostate cancer diagnosis by volatile metabolic profiling of urine. Br. J. Cancer.

[107] Wishart D.S., Feunang Y.D., Marcu A., Guo A.C., Liang K., Vázquez-Fresno R., Sajed T., Johnson D., Li C., Karu N. (2018). HMDB 4.0: The human metabolome database for 2018. Nucleic Acids Res..

[108] Jia Z., Patra A., Kutty V.K., Venkatesan T. (2019). Critical Review of Volatile Organic Compound Analysis in Breath and In Vitro Cell Culture for Detection of Lung Cancer. Metabolites.

[109] Owen O.E., Trapp V.E., Skutches C.L., Mozzoli M.A., Hoeldtke R.D., Boden G., Reichard G.A. (1982). Acetone Metabolism during Diabetic Ketoacidosis. Diabetes.

[110] Alpay Savasan Z., Yilmaz A., Ugur Z., Aydas B., Bahado-Singh R.O., Graham S.F. (2019). Metabolomic Profiling of Cerebral Palsy Brain Tissue Reveals Novel Central Biomarkers and Biochemical Pathways Associated with the Disease: A Pilot Study. Metabolites.

[111] Wang C., Sun B., Guo L., Wang X., Ke C., Liu S., Zhao W., Luo S., Guo Z., Zhang Y. (2014). Volatile Organic Metabolites Identify Patients with Breast Cancer, Cyclomastopathy, and Mammary Gland Fibroma. Sci. Rep..

[112] Guilherme A., Virbasius J.V., Puri V., Czech M.P. (2008). Adipocyte dysfunctions linking obesity to insulin resistance and type 2 diabetes. Nat. Rev. Mol. Cell Bio..

[113] American Diabetes Association Professional Practice Committee (2022). 2. Classification and Diagnosis of Diabetes: Standards of Medical Care in Diabetes—2022. Diabetes Care.

[114] Scott D., Renaud D., Krishnasamy S., Meriç P., Buduneli N., Çetinkalp C., Liu K. (2010). Diabetes-related molecular signatures in infrared spectra of human saliva. Diabetol. Metab. Syndr..

[115] Saasa V., Malwela T., Beukes M., Mokgotho M., Liu C.P., Mwakikunga B. (2018). Sensing Technologies for Detection of Acetone in Human Breath for Diabetes Diagnosis and Monitoring. Diagnostics.

[116] Trefz P., Obermeier J., Lehbrink R., Schubert J.K., Miekisch W., Fischer D.C. (2019). Exhaled volatile substances in children suffering from type 1 diabetes mellitus: Results from a cross-sectional study. Sci. Rep..

[117] Wang Z., Wang C. (2013). Is breath acetone a biomarker of diabetes? A historical review on breath acetone measurements. J. Breath Res..

[118] Storer M., Dummer J., Lunt H., Scotter J., McCartin F., Cook J., Swanney M., Kendall D., Logan F., Epton M. (2011). Measurement of breath acetone concentrations by selected ion flow tube mass spectrometry in type 2 Diabetes. J. Breath Res..

[119] Tuzson B., Looser H., Felder F., Bovey F., Tappy L., Emmenegger L. (2018). Human Breath Acetone Analysis by Mid-IR Laser Spectroscopy: Development and Application. Proceedings of the High-Brightness Sources and Light-Driven Interactions.

[120] Reyes-Reyes A., Horsten R.C., Urbach H.P., Bhattacharya N. (2015). Study of the Exhaled Acetone in Type 1 Diabetes Using Quantum Cascade Laser Spectroscopy. Anal. Chem..

[121] Johnson T.J., Sams R.L., Sharpe S.W., Sedlacek A.J., Colton R., Vo-Dinh T. (2004). The PNNL quantitative infrared database for gas-phase sensing: A spectral library for environmental, hazmat, and public safety standoff detection. Chemical and Biological Point Sensors for Homeland Defense.

[122] Apolonski A., Maiti K.S. (2021). Towards a standard operating procedure for revealing hidden volatile organic compounds in breath: The Fourier-transform IR spectroscopy case. Appl. Opt..

[123] Prabhakar A., Quach A., Zhang H., Terrera M., Jackemeyer D., Xian X., Tsow F., Tao N., Forzani E.S. (2015). Acetone as biomarker for ketosis buildup capability—A study in healthy individuals under combined high fat and starvation diets. Nutr. J..

[124] Alfarouk K.O., Bashir A.H.H., Aljarbou A.N., Ramadan A.M., Muddathir A.K., AlHoufie S.T.S., Hifny A., Elhassan G.O., Ibrahim M.E., Alqahtani S.S. (2019). The Possible Role of Helicobacter pylori in Gastric Cancer and Its Management. Front. Oncol..

[125] Scanu T., Spaapen R.M., Bakker J.M., Pratap C.B., Wu L.E., Hofland I., Broeks A., Shukla V.K., Kumar M., Janssen H. (2015). Salmonella Manipulation of Host Signaling Pathways Provokes Cellular Transformation Associated with Gallbladder Carcinoma. Cell Host Microbe.

[126] Mager D. (2006). Bacteria and cancer: Cause, coincidence or cure? A review. J. Transl. Med..

[127] Traulsen J., Zagami C., Daddi A.A., Boccellato F. (2021). Molecular modelling of the gastric barrier response, from infection to carcinogenesis. Best Pract. Res. Clin. Gastroenterol..

[128] Hall K.K., Lyman J.A. (2006). Updated Review of Blood Culture Contamination. Clin. Microbiol. Rev..

[129] Boyles T.H., Wasserman S. (2015). Diagnosis of bacterial infection. SAMJ S. Afr. Med. J..

[130] Peri A.M., Stewart A., Hume A., Irwin A., Harris P.N.A. (2021). New Microbiological Techniques for the Diagnosis of Bacterial Infections and Sepsis in ICU Including Point of Care. Curr. Infect. Dis. Rep..

[131] Váradi L., Luo J.L., Hibbs D.E., Perry J.D., Anderson R.J., Orenga S., Groundwater P.W. (2017). Methods for the detection and identification of pathogenic bacteria: Past, present, and future. Chem. Soc. Rev..

[132] Maiti K.S., Apolonski A. (2021). Monitoring the Reaction of the Body State to Antibiotic Treatment against Helicobacter pylori via Infrared Spectroscopy: A Case Study. Molecules.

[133] Doig P., de Jonge B.L., Alm R.A., Brown E.D., Uria-Nickelsen M., Noonan B., Mills S.D., Tummino P., Carmel G., Guild B.C. (1999). *Helicobacter pylori* Physiology Predicted from Genomic Comparison of Two Strains. Microbiol. Mol. Biol. Rev..

[134] Blair E., Watson L. (2006). Epidemiology of cerebral palsy. Semin. Fetal Neonatal Med..

[135] Patel D.R., Neelakantan M., Pandher K., Merrick J. (2020). Cerebral palsy in children: A clinical overview. Transl. Pediatr..

[136] Wimalasundera N., Stevenson V.L. (2016). Cerebral palsy. Pract. Neurol..

[137] Roy S., Alves-Pinto A., Lampe R. (2018). Modeling of Muscle Activation from Electromyography Recordings in Patients with Cerebral Palsy. Appl. Sci..

[138] Roy S., Alves-Pinto A., Lampe R. (2018). Characteristics of Lower Leg Muscle Activity in Patients with Cerebral Palsy during Cycling on an Ergometer. BioMed Res. Int..

[139] Lampe R., Botkin N., Turova V., Blumenstein T., Alves-Pinto A. (2014). Mathematical Modelling of Cerebral Blood Circulation and Cerebral Autoregulation: Towards Preventing Intracranial Hemorrhages in Preterm Newborns. Comput. Math. Methods Med..

[140] Maiti K.S., Roy S., Lampe R., Apolonski A. (2019). Breath signatures of cerebral palsy patients revealed with mid-infrared FTIR spectroscopy. Proceedings of the 2019 Conference on Lasers and Electro-Optics Europe and European Quantum Electronics Conference.

[141] Maiti K.S., Roy S., Lampe R., Apolonski A. (2020). Breath indeed carries significant information about a disease. Potential biomarkers of cerebral palsy. J. Biophotonics.

[142] Lewis P., Lewis K., Ghosal R., Bayliss S., Lloyd A.J., Wills J., Godfrey R., Kloer P., Mur L.A.J. (2010). Evaluation of FTIR Spectroscopy as a diagnostic tool for lung cancer using sputum. BMC Cancer.

[143] Bird B., Miljković M., Remiszewski S., Akalin A., Kon M., Diem M. (2012). Infrared spectral histopathology (SHP): A novel diagnostic tool for the accurate classification of lung cancer. Lab Investig..

[144] Gashimova E., Temerdashev A., Porkhanov V., Polyakov I., Perunov D., Azaryan A., Dmitrieva E. (2020). Investigation of different approaches for exhaled breath and tumor tissue analyses to identify lung cancer biomarkers. Heliyon.

[145] Long Y., Wang C., Wang T., Li W., Dai W., Xie S., Tian Y., Liu M., Liu Y., Peng X. (2020). High performance exhaled breath biomarkers for diagnosis of lung cancer and potential biomarkers for classification of lung cancer. J. Breath Res..

[146] Amann A., Corradi M., Mazzone P., Mutti A. (2011). Lung cancer biomarkers in exhaled breath. Expert Rev. Mol. Diagn..

[147] Campanella A., Summa S.D., Tommasi S. (2019). Exhaled breath condensate biomarkers for lung cancer. J. Breath Res..

[148] Marchand L.L., Wilkens L.R., Harwood P., Cooney R.V. (1993). Breath hydrogen and methane in populations at different risk for colon cancer. Int. J. Cancer.

[149] Kim Y.J., Kim W.J. (2016). Can we use methylation markers as diagnostic and prognostic indicators for bladder cancer?. Investig. Clin. Urol..

[150] Pentyala S., Whyard T., Pentyala S., Muller J., Pfail J., Parmar S., Helguero C.G., Khan S. (2016). Prostate cancer markers: An update (Review). Biomed. Rep..

[151] Khalid T., Aggio R., White P., De Lacy Costello B., Persad R., Al-Kateb H., Jones P., Probert C.S., Ratcliffe N. (2015). Urinary Volatile Organic Compounds for the Detection of Prostate Cancer. PLoS ONE.

[152] Rawla P. (2019). Epidemiology of Prostate Cancer. World J. Oncol..

[153] Gomella L., Liu X., Trabulsi E., Kelly W., Myers R., Showalter T., Dicker A., Wender R. (2011). Screening for Prostate Cancer: The Current Evidence and Guidelines Controversy. Can. J. Urol..

[154] Huang Y., Li Z.Z., Huang Y.L., Song H.J., Wang Y.J. (2018). Value of free/total prostate-specific antigen (f/t PSA) ratios for prostate cancer detection in patients with total serum prostate-specific antigen between 4 and 10 ng/mL. Medicine.

[155] Cornu J.N., Cancel-Tassin G., Ondet V., Girardet C., Cussenot O. (2011). Olfactory Detection of Prostate Cancer by Dogs Sniffing Urine: A Step Forward in Early Diagnosis. Eur. Urol..

[156] Pirrone F., Albertini M. (2017). Olfactory detection of cancer by trained sniffer dogs: A systematic review of the literature. J. Vet. Behav..

[157] Maiti K.S., Fill E., Strittmatter F., Volz Y., Sroka R., Apolonski A. (2021). Accurate diagnosis of prostate cancer via infrared spectroscopy of breath. Proceedings of the European Conferences on Biomedical Optics 2021 (ECBO).

[158] Maiti K.S., Fill E., Strittmatter F., Volz Y., Sroka R., Apolonski A. (2021). Towards reliable diagnostics of prostate cancer via breath. Sci. Rep..

[159] Muraviev A.V., Smolski V.O., Loparo Z.E., Vodopyanov K.L. (2018). Massively parallel sensing of trace molecules and their isotopologues with broadband subharmonic mid-infrared frequency combs. Nat. Photonics.

[160] Pupeza I., Hofer C., Gerz D., Fürst L., Högner M., Butler T., Gebhardt M., Heuermann T., Gaida C., Maiti K. (2022). Field-resolved spectroscopy approaching ultimate detection sensitivity. Res. Sq..

[161] Selvaraj R., Vasa N.J., Nagendra S.M.S., Mizaikoff B. (2020). Advances in Mid-Infrared Spectroscopy-Based Sensing Techniques for Exhaled Breath Diagnostics. Molecules.

[162] Liang Q., Chan Y.C., Changala P.B., Nesbitt D.J., Ye J., Toscano J. (2021). Ultrasensitive multispecies spectroscopic breath analysis for real-time health monitoring and diagnostics. Proc. Natl. Acad. Sci. USA.

[163] Naz F., Groom A.G., Mohiuddin M., Sengupta A., Daigle-Maloney T., Burnell M.J., Michael J.C.R., Graham S., Beydaghyan G., Scheme E. (2022). Using infrared spectroscopy to analyze breath of patients diagnosed with breast cancer. J. Clin. Oncol..

[164] Röck F., Barsan N., Weimar U. (2008). Electronic Nose: Current Status and Future Trends. Chem. Rev..

[165] Anisimov D.S., Chekusova V.P., Trul A.A., Abramov A.A., Borshchev O.V., Agina E.V., Ponomarenko S.A. (2021). Fully integrated ultra-sensitive electronic nose based on organic field-effect transistors. Sci. Rep..

